# New model of proliferative vitreoretinopathy in rabbit for drug delivery and pharmacodynamic studies

**DOI:** 10.1080/10717544.2018.1440664

**Published:** 2018-02-20

**Authors:** Sang Woong Moon, Yaoyao Sun, David Warther, Kristyn Huffman, William R. Freeman, Michael J. Sailor, Lingyun Cheng

**Affiliations:** a Department of Ophthalmology, Jacobs Retina Center at Shiley Eye Institute, University of California San Diego, La Jolla, CA, USA;; b Department of Ophthalmology, College of Medicine, Kyung Hee University, Seoul, Republic of Korea;; c Department of Ophthalmology, Ophthalmology & Optometry Center, Peking University People’s Hospital, Beijing, China;; d Department of Chemistry and Biochemistry, University of California San Diego, La Jolla, CA, USA

**Keywords:** Intravitreal drug delivery, porous silicon, dexamethasone, rabbit model of PVR, VEGF, Matrigel, subretinal injection, OCT, fundus fluorescein angiography

## Abstract

Blinding retinal diseases become more epidemic as the population ages. These diseases, such as diabetic retinopathy and macular edema, are of chronic nature and require protracted drug presence at the disease site. A sustained intravitreal porous silicon delivery system with dexamethasone (pSiO_2_-COO-DEX) was evaluated in a new rabbit model of proliferative vitreoretinopathy (PVR) in a real treatment design. In contrast to the pretreatment design model, pSiO_2_-COO-DEX was intravitreally injected into the eyes with active inflammation. Subretinal injection of vascular endothelial growth factor (VEGF) and Matrigel induced a late-onset vitreoretinal inflammation that gradually developed into PVR. This method mimics the human disease better than PVR induced by either intravitreal cell injection or trauma. The pSiO_2_-COO-DEX intervened eyes had minimal PVR, while balanced saline solution or free dexamethasone intervened eyes had significantly more PVR formation. In addition, adding VEGF to the Matrigel for subretinal injection induced greater inflammation and retinal neovascularization in comparison to only Matrigel injected under the medullary ray. Clinical and pathological examinations, including fundus fluorescein angiography and optical coherence tomography, confirmed these changes. In the current study, neither subretinal injection of Matrigel or subretinal injection of VEGF and Matrigel induced choroidal neovascularization. However, the current PVR model demonstrates a chronic course with moderate severity, which may be useful for drug screening studies.

## Introduction

Retinal and choroidal neovascularization (CNV) occur in several blinding diseases such as exudative age-related macular degeneration, diabetic retinopathy, and retinopathy of prematurity. Our current knowledge indicates that these phenomena result from over-expressed vascular endothelial growth factor (VEGF) as well as persistent low-grade inflammation (Agawa et al., 2014; Arimura et al., 2016; Campochiaro et al., 2016; Liu et al., 2016; Rezar-Dreindl et al., 2016). Anti-VEGF agents are now widely used as the main pharmacological treatment modality for these refractory retinal diseases (Wang et al., 2015; Xu & Tan, 2017). Anti-VEGF treatment can effectively neutralize over-expressed VEGF, however, these monoclonal antibodies usually do not possess anti-inflammatory properties (Arimura et al., 2016).

Dexamethasone (DEX) is a glucocorticoid widely used in ophthalmology, despite the risk of ocular side effects, such as elevated intraocular pressure (IOP) and cataract formation. The recently FDA approved dexamethasone implant (Ozurdex) has been employed for the treatment of macular edema from retinal vein occlusion and diabetic retinopathy, although ocular side effects are of concern to patients and physicians (Meyer & Schonfeld, 2013; Ramu et al., 2015; Bakri et al., 2016). Ozurdex intravitreal injection is beneficial in treating macular edema. However, a recent clinical trial revealed that Ozurdex did not provide preventative effects against proliferative retinopathy (PVR) reoccurrence (Banerjee et al., 2017), a common blinding eye disease developing from diabetic retinopathy, ocular trauma, and retinal detachment. This highlights the complexity of the pharmacodynamics of each retinal disease and its management. In contrast with macular edema, PVR presents with profuse cell proliferation and retinal neovascularization. To suppress this pathogenesis, the drug level and drug dynamics from Ozurdex may not be ideal.

We have previously reported a porous silicon (pSi) based DEX delivery system (pSiO_2_-COO-DEX) (Wang et al., 2014b) that has demonstrated good intraocular biocompatibility (Cheng et al., 2008) and is biodegradable into various protonated forms of the orthosilicate ion to be cleared from the eye (Nieto et al., 2013; Hou et al., 2014). This pSiO_2_-COO-DEX delivery system is administered by a smaller needle than Ozurdex and more importantly, DEX-loaded pSi particles are placed in the posterior vitreous closer to the disease site (macula), while Ozurdex is placed at the vitreous base close to the anterior chamber and lens. Ocular side effects may be more likely with an implant placed near the anterior section of the eye. In fact, implant migration into the anterior chamber has been reported to cause cornea damage (Pardo-Lopez et al., 2012; El-Ghrably et al., 2015). The pSi-based DEX delivery system has demonstrated good pretreatment efficacy on a mild, VEGF-induced, retinal vascular permeability model in rabbits (Hou et al., 2016). In that model, human VEGF was injected into the vitreous to induce breakdown of the retinal blood barrier but did not include an inflammation element and the blood vessel leakage was transient. PVR is a more severe disease entity than the VEGF-induced vitritis. Traditional PVR models are induced either through intravitreal injection of exogenous cells or via trauma and intravitreal blood injection, which precludes objective clinical observation and grading. In addition, these models develop too fast and deviate too much from the PVR seen in human eyes (Cheng et al., 2010; Hou et al., 2015). The current study was designed to evaluate the efficacy of a pSi-based DEX delivery system on a new PVR model that has the pathological hallmarks of proliferation and neovascularization as well as a slow progressing nature.

## Methods and materials

### Experimental materials

Dexamethasone, 3-aminopropyltrimethoxysilane, succinic anhydride, ethanol, 4-dimethylaminopyridine, dicyclohexylcarbodiimide (DCC), N,N-dimethylformamide, and hydrochloric acid were purchased from Sigma-Aldrich (St. Louis, MO, USA). Ethanol was purchased from Thermo Fisher Scientific (Waltham, MA, USA). (100)-Oriented boron-doped p-type silicon wafers were purchased from Siltronix Inc. (0.99 mΩ • cm resistivity, Archamps, France). Matrigel basement membrane matrix (REF 356234, Corning, Corning, NY, USA) was thawed at 4 °C overnight before use. Human VEGF (Sigma, St. Louis, MO, USA) was reconstituted in 1% bovine serum albumin in sterile phosphate-buffered saline at 0.5 μg/μL as stock solution and suspended in Matrigel at 750 ng per 20 μL.

### Dexamethasone loaded porous silicon microparticles (pSiO2-COO-DEX)

Porous silicon microparticles were prepared by electrochemical etching of silicon wafers in a 48% aqueous hydrofluoric acid:ethanol (3:1 by volume) electrolyte solution. The resulting porous layer was then lifted off by electropolishing in a 1:29 solution of 48% aqueous hydrofluoric acid to ethanol. The etched porous layers were ultrasonicated in ethanol for 30 minutes to form the microparticles as we reported previously (Wang et al., 2014a). For loading of dexamethasone, surface functionalized particles (pSiO_2_-COOH) were mixed with DCC (13 mg), 4-N,N-dimethylamminopyridine (3 mg), dichloromethane (DCM, 1.2 mL), and dexamethasone (4 mg). The mixture was rotated for seven days at room temperature. Particles were separated by centrifugation and carefully washed with DCM and ethanol to remove unloaded drug and other chemicals before they were dried for intravitreal injection (Hou et al., 2014; Wang et al., 2014a).

### In vivo study design

Although laser-induced rodent CNV has elements of both proliferation and neovascularization, rat eyes deviate too much from human eyes and have a very limited volume of vitreous (40–50 µL) (Sha & Kwong, 2006–07), which is not ideal for evaluating a sustained vitreous drug delivery system. Rabbits have a much larger vitreous volume (1200–1500 µL) than that of the rat and have been used to develop CNV models by subretinal injection of growth factors impregnated in Matrigel or gelatin (Kimura et al., 1995; Browning et al., 2005; Qiu et al., 2006). Qiu et al. reported Matrigel alone induced the same rate of CNV as VEGF impregnated Matrigel (Qiu et al., 2006). It seems that CNV is likely attributed to the Matrigel rather than to the exogenous VEGF. Matrigel is extracted from a mouse sarcoma and consists of laminin, collagen IV, entactin, heparan sulfate proteoglycan, and growth factors. We hypothesized that subretinal Matrigel with or without VEGF may cause a retinal inflammation and neovascularization disease such as PVR. We suspect that since the injected inflammatory and angiogenesis agents in the viscous Matrigel are trapped under the retina, the disease course could have a chronic nature. In the Qiu et al. study, Matrigel or VEGF-impregnated Matrigel was injected under the rabbit’s medullary ray. The rabbit retina does not have a macula like humans; however, there is an equivalent location called the visual streak at which photoreceptor and ganglion cells have the highest density and a high demand for oxygen. This site was elected for subretinal injection of Matrigel, in addition to the medullary ray to repeat the previously published study.

To carefully characterize the retinal pathological changes over time, clinical evaluations were our primary focus due to the nature of this preclinical work. Instead of employing many animals, we used a small number of animals with more rigorous examination modalities at many time points to collect detailed longitudinal data. This meticulous approach maximizes the information obtained while minimizing the number of animals used and the associated costs of keeping large cohorts of animals for an extended period.

For this study, a total of nine pigmented New Zealand rabbits were used (mean body weight of 4.2 kg). All animal procedures performed in this study adhere to the provisions of the ARVO Statement for the Use of Animals in Ophthalmic and Vision Research, and were approved by the Institutional Animal Care and Use Committee of the University of California, San Diego. The animals were anesthetized with a subcutaneous injection of 25 mg/kg ketamine (Fort Dodge Animal Health, Fort Dodge, IA, USA) and 4 mg/kg xylazine (Akorn Inc., Decatur, IL, USA). Only one eye of each animal was used. The pupil was dilated with 1% tropicamide (Akorn Inc., Decatur, IL, USA) and 2.5% phenylephrine hydrochloride (Akorn Inc., Decatur, IL, USA). 0.5% proparacaine hydrochloride (Akorn Inc., Decatur, IL, USA) was used as topical anesthetic. Under a surgical microscope, a sclerotomy was made 1.5 mm posterior to the corneal limbus in the supranasal quadrant using a 20-gauge micro vitreoretinal blade. A 20-gauge cannula tapered to a 32-gauge angled cannula (REF 171-32, Eagle Labs, Rancho Cucamonga, CA, USA) was attached to 6-inch long tubing (Ocular Irrigation Tube, Eagle Labs, Rancho Cucamonga, CA, USA) that was attached to a 250 µL Hamilton glass syringe (Hamilton Co., Reno, NV, USA). The Hamilton syringe was loaded onto a repeating dispenser (Hamilton Co., Reno, NV, USA) with which each actuation delivers 1/50 of the total capacity of that syringe. The cannula was introduced through the sclerotomy into the vitreous cavity. After the 32-gauge cannula tip was positioned under the retina, 20 µL of Matrigel (with or without VEGF) was injected. The injection resulted in a localized subretinal bleb/neurosensory retinal detachment. After the procedure, 0.5% topical moxifloxacin eye drops (Alcon Laboratories, Fort Worth, TX, USA) were given once a day for three days. No steroid eye drops were used.

#### Subretinal injection at the medullary ray

**Figure 1.**
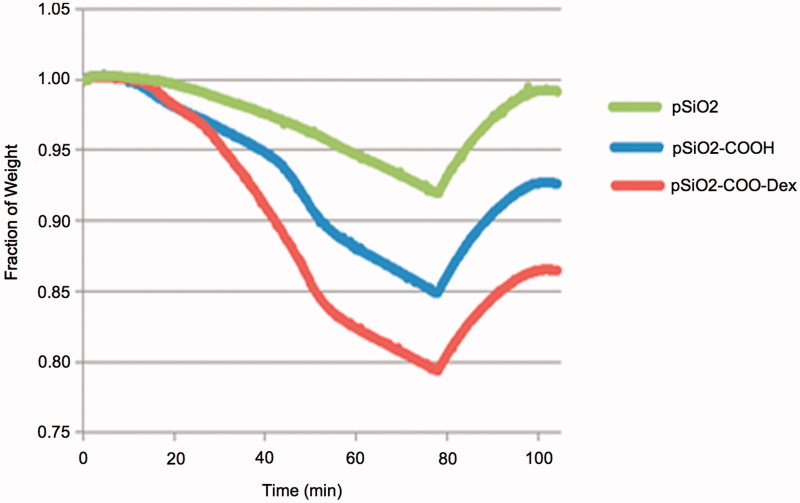
TGA analysis. Evolution of mass of the samples over time (19.8 mg of dry particles). pSiO_2_: bare oxidized particles; pSiO_2_-COOH: particles grafted with linker (free COOH function); pSiO_2_-COO-Dex: particles loaded with dexamethasone.

Three rabbits were used for this route of injection and only their right eyes were injected. Twenty microliters of Matrigel was injected and a dome-shaped detachment was recognizable (Supplemental Figure 1^Figure 1.^). After the injection, these three rabbits were followed up without any intervention to follow the natural course of clinical manifestation and terminal histology.

#### Subretinal injection at the visual streak

Six rabbits received subretinal injections of 20 µL VEGF/Matrigel suspension (750 ng VEGF) in their right eyes. Two subretinal blebs, neighboring each other, were created by two separate 10 µL injections (Supplemental Figure 2) in the visual streak, 2–3 mm inferior to the optic nerve disc. The two injection sites were two optic disc diameters apart.

**Figure 2.**
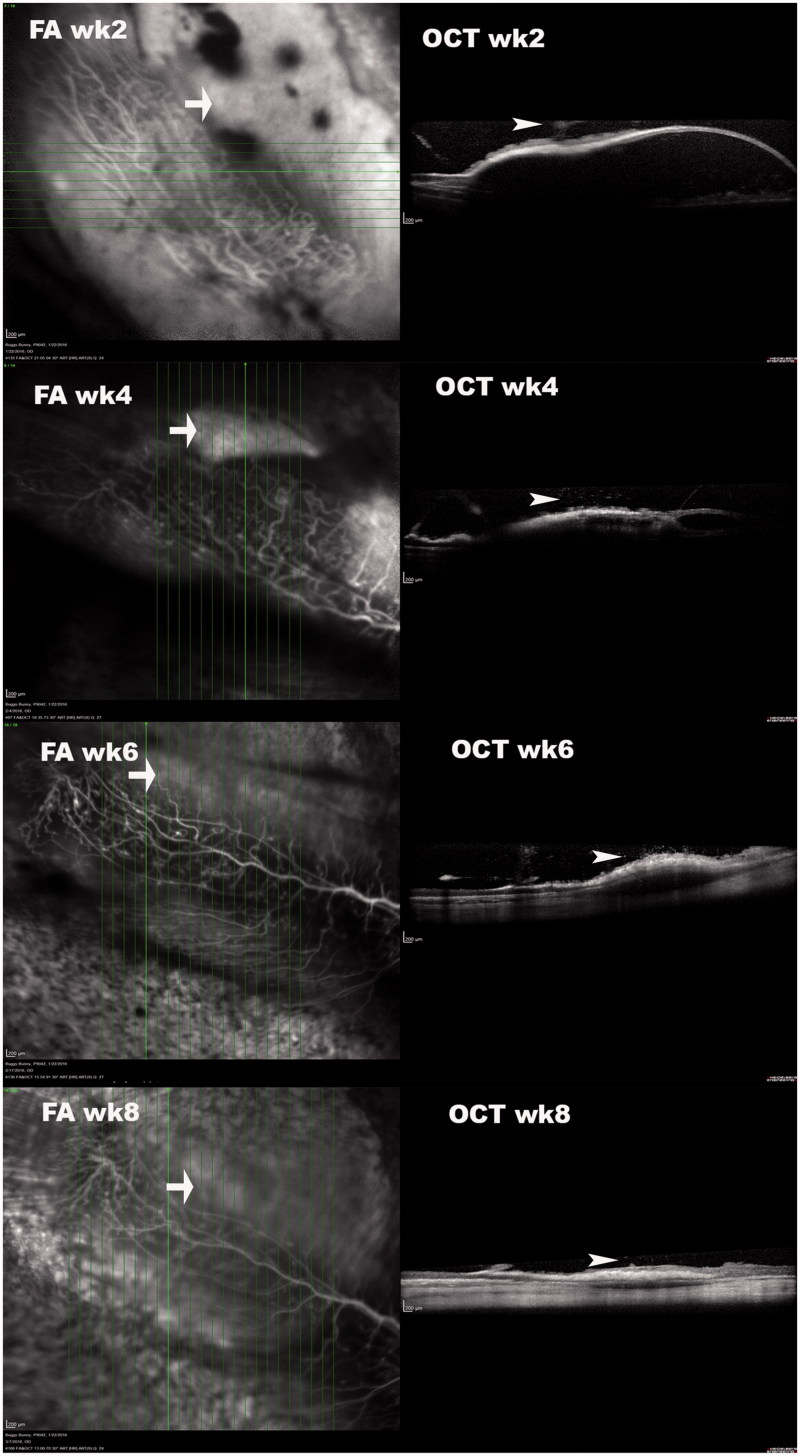
OCT of subretinally injected Matrigel at the medullary ray. These simultaneous FA and OCT images at different time points were acquired 15–20 minutes after intravenous injection of sodium fluorescein. OCT demonstrated that the retinal detachments and subretinal fluid diminished over time. The sodium fluorescein pooling into the subretinal fluid was recognizable (arrow) and decreased in area and intensity over time. It did not completely disappear by week 8. The vitreous opacity or granules (arrowhead) immediately above the detached retina were recognizable in OCT frames, indicating inflammation.

#### Pharmacological interventions

Two weeks after subretinal injection of the VEGF/Matrigel suspension, the rabbits injected at the visual streak received drug treatment or control injection. Two rabbits had their right eyes injected with 3 mg of pSiO_2_-COO-DEX suspended in 100 µL balanced salt solution (BSS) as the study group. Two other rabbits had their right eyes injected with a dose of free dexamethasone in 100 µL BSS, equal to the mass of DEX in 3 mg of pSiO_2_-COO-DEX. This group served as a free drug control. The last two rabbits received an intravitreal injection of 100 µL BSS and served as the control. All injections were performed using a 1 mL syringe and 27-gauge needle under direct view of a surgical microscope while the animal was under general anesthesia.

#### Examinations and follow-up

Following the subretinal Matrigel injection, indirect ophthalmoscopy and slit-lamp biomicroscopy were performed on post-injection days 3, 7, 14, and 21, at which time, the pharmacological intervention was conducted. Slit-lamp biomicroscopy and fundus imaging (Canon EOS Rebel T2i; Canon Inc., Tokyo, Japan) were performed every week thereafter. Fluorescein angiography (FA) and optical coherence tomography (OCT, Spectralis, Heidelberg Engineering, Heidelberg, Germany) were done two weeks after the subretinal injection of the Matrigel and then every two weeks after the pharmacological intervention up to 11 weeks. At each exam, the aqueous was graded by inflammatory cell counts delineated by the Standardization of Uveitis Nomenclature (SUN) Working Group (Jabs et al., 2005) and the vitreous was graded for clarity, which is closely associated with the extent of vitritis. Vitreous clarity was graded from 0 to 8 according to the Photographic Vitreous Haze Grading Technique for Clinical Trials in Uveitis reported by Madow et al. (Madow et al., 2011). For PVR grading, a slightly modified criterion from the published scales (developed for the rabbit PVR model from intravitreally injected homologous retinal pigment epithelial (RPE) cells) (Agrawal et al., 2007) was used to better reflect the nature of the observed proliferation. In this grading system, grade 1 = vitreous haze, grade 2 = vitreous membrane, grade 3 = elevation of one medullary ray, grade 4 = elevation of both medullary rays, grade 5 = medullary elevation and retinal neovascularization, grade 6 = retinal detachment around the elevated medullary rays, and grade 7 = extensive retinal detachment involving the posterior pole. After sacrifice, the eye globes were enucleated and fixed in 10% formalin for histological evaluation. Paraffin-embedded tissue sections were produced for hematoxylin and eosin (H&E) staining and light microscopy.

#### Statistical analysis

In this study, clinical aqueous humor inflammation grading, vitreous clarity grading, and PVR grading were conducted at multiple times during the disease course from the same eyes. For statistical analysis, clinical inflammation scores were pooled from the aqueous inflammation and vitreous inflammation gradings. The pooled inflammation or PVR scores were used as dependent response and the group as independent variable along with the covariable of timing (at which inflammation or PVR was graded) in a GEE (generalized estimating equation) regression model with multinomial distribution and link function of cumulative logit in SAS environment (SAS version 9.4 Cary, NC: SAS Institute Inc.).

## Results

### pSi particle characteristics and drug loading efficiency

The particle size was measured under a light microscope from a sample of particles following oxidation and before drug loading. The mean particle size was 29 ± 10 µm by 29 ± 11 µm. The amount of drug loaded into the particles was measured by thermogravimetric analysis (TGA). The mass of the linker represents 6.58% (percent weight loss of pSiO_2_-COOH minus pSiO_2_) and the mass of DEX represents 6.16% of the mass of the particles (percent weight loss of pSiO_2_-COO-DEX minus pSiO_2_-COOH) (Figure 1).

### Inflammatory course of subretinally injected matrigel at the medullary ray

After injection, the eyes were quiet during the first week, then vitritis gradually appeared at the injection site within the second week. Vitreous inflammation increased and expanded into the rest of the vitreous along with the development of medullary ray distortion. Vitreous inflammation reached its peak at weeks 4 and 5 before decreasing. From week 6 to week 8, the inflammation receded back to the injection site and finally disappeared, leaving behind wrinkling or distortion of the medullary rays and a bleb shaped chorioretinal atrophy (Supplemental Figure 3).

**Figure 3.**
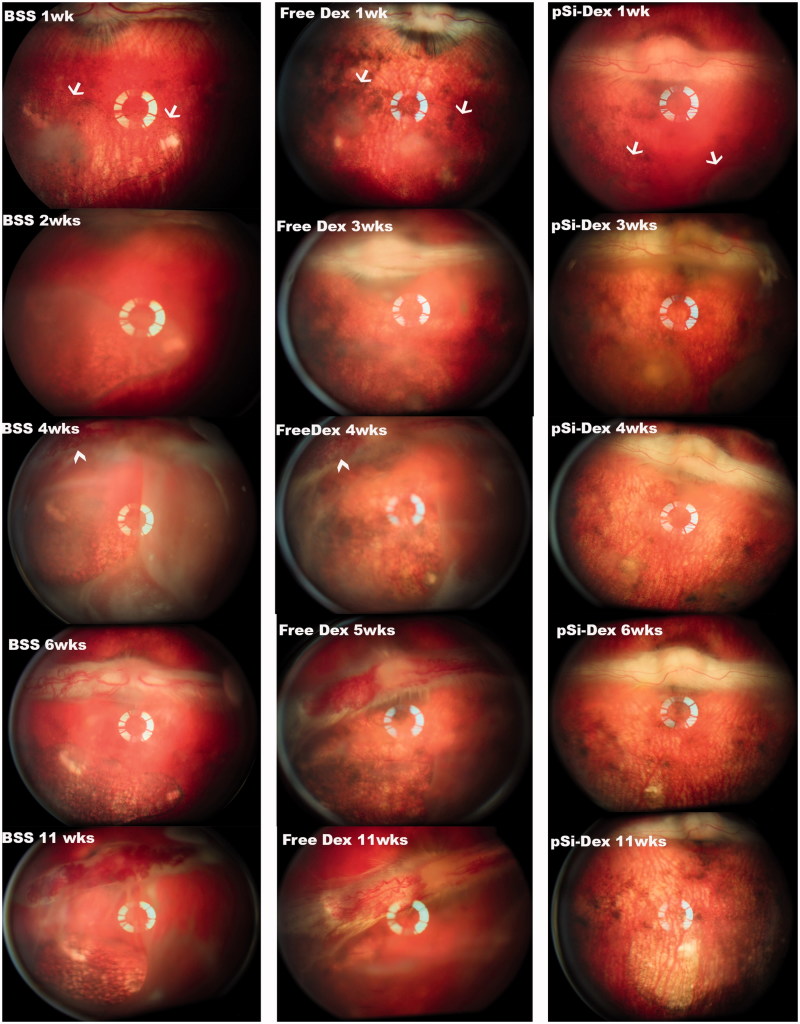
pSiO_2_-COO-DEX treatment vs. controls. The images of the left column are from a control eye (BSS injection); the images in the middle column are from one of the free Dex injected eyes; and the images of the right column are from an eye treated by intravitreal pSiO_2_-COO-DEX (pSi-Dex). All eyes were relatively quiet during the first week after subretinal VEGF/Matrigel injection and the clear vitreous allows identification of the two subretinal blebs (arrows) on the color fundus images. After pharmacological intervention, severe vitritis emerged in the eyes injected with BSS or free Dex. Retinal neovascularization on the medullary ray (arrowheads) was spotted during week 4 and persisted until the end of the study. In contrast, vitritis was mild and no neovascularization was noted on the medullary ray in the eyes injected with pSiO_2_-COO-DEX (far right column).

Images during early phase, mid phase, and late phase FA demonstrated hypofluorescence in the early phase (Supplemental Figure 4, top left) and pooling of fluorescence into the subretinal fluid in the late phase (Supplemental Figure 4, bottom left). No characteristic leaking from choroidal neovascularization was noted. FA images from approximately 5 min after the injection of fluorescein dye were assembled in Supplemental Figure 5 from several different time points. Those images did not reveal CNV either. Figure 2 demonstrated the simultaneous FA and OCT findings of the lesions from subretinal injection of Matrigel at the medullary ray. From the very late phase (15 min to 20 min) of FA and corresponding OCT images, it was clear that the subretinal pooling of fluorescein was corresponding to where serous retinal detachment had occurred as shown on OCT. Histology under light microscopy of H&E stained paraffin sections did not reveal neovascularization between the damaged RPE layer and the outer nuclear layer.

**Figure 4.**
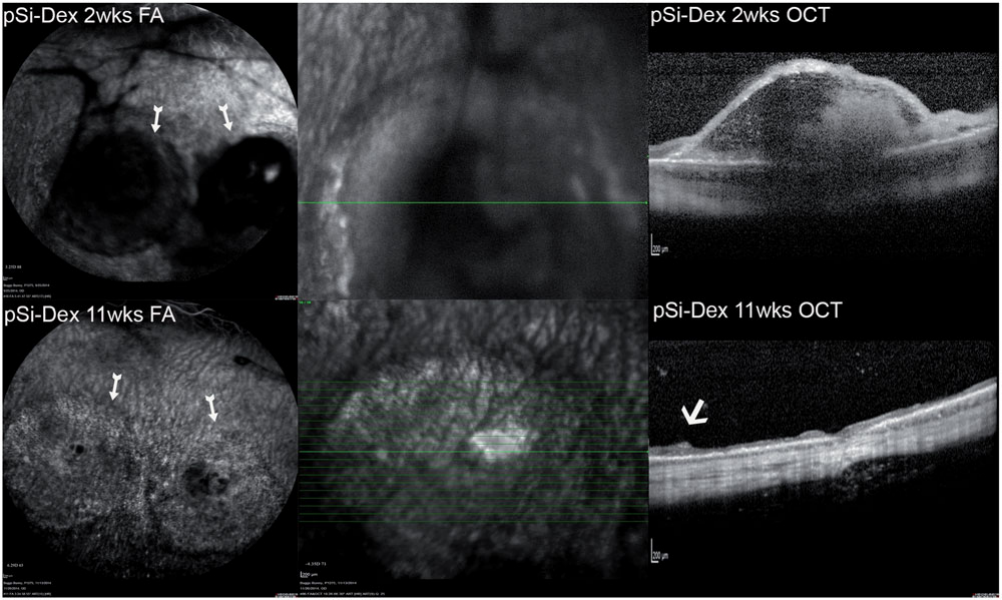
pSiO_2_-COO-DEX treated eye FA and OCT. FA image in the upper left column, two weeks after the subretinal VEGF/Matrigel injection, shows two round areas of hypofluorescence corresponding to the subretinal injections (arrows). Subretinal Matrigel blocked the fluorescence from the underlying choroid. Vitritis is seen here as black strands in the vitreous, blocking the fluorescence from the choroid underneath. The OCT image in the upper right column demonstrates a dome-shaped retinal bleb with trapped Matrigel within. The lower panel is from the same eye 11 weeks after Matrigel injection or 8 weeks after pSiO_2_-COO-DEX (pSi-Dex) intravitreal injection. The FA image shows clear vitreous and two round areas of uneven fluorescence corresponding to the atrophic retinal blebs (arrow). The OCT image on the right shows retinal thinning at the bleb area. The arrow points the edge of the atrophic area.

**Figure 5.**
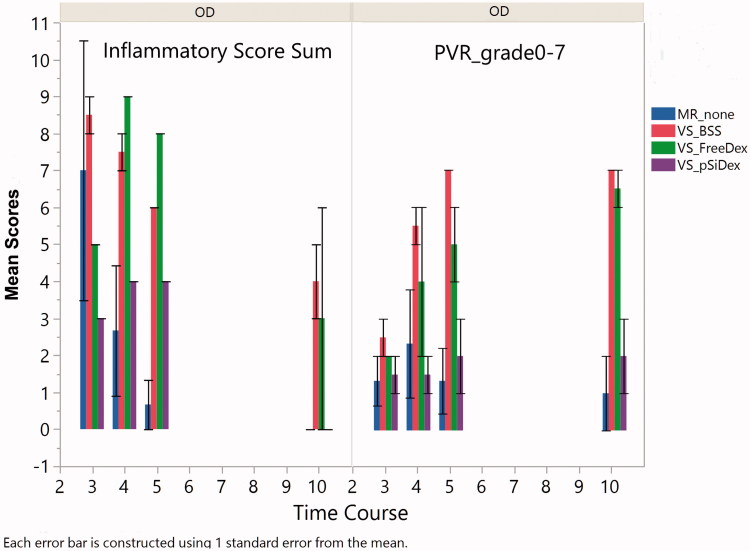
Inflammation and PVR scores. The left panel bar graph demonstrates total inflammation in aqueous and vitreous for eyes injected at the medullary ray (MR) or visual streak (VS) with different formulations of intervention. In general, the inflammation scores descended over time. The eyes with Matrigel + VEGF injection at the visual streak (VS) had a stronger inflammatory reaction compared to the eyes injected with Matrigel only under the medullary ray. The right panel bar graph demonstrates significantly more PVR in the eyes injected with Matrigel + VEGF and intervened with intravitreal BSS or free Dex as compared with the eyes treated with intravitreal pSiO_2_-COO-DEX (VS_pSiDex).

### Inflammatory course from subretinal injection of VEGF/matrigel at visual streak and pharmacological intervention

Six rabbits received a subretinal injection of VEGF/Matrigel suspension at the visual streak of their right eyes. The intravitreal intervention was conducted three weeks after the injection of VEGF/Matrigel. Both indirect ophthalmoscopy and fundus fluorescein angiography showed that free DEX did not suppress the vitreous inflammation. The time course of the inflammation was similar to the eyes injected with BSS (Figure 3, *p* = .59, GEE). These eyes showed obvious vitritis at week 2 following the injection of VEGF/Matrigel and the inflammation spread further in week 4, showing the start of retinal neovascularization on the medullary ray. The vitreous haze did not completely resolve until week 11. Retinal neovascularization presented by week 4 and persisted until the end of the study (Figure 3, BSS 11 wks and free DEX 11 wks). In contrast to the eyes injected with BSS or free dexamethasone, the eyes injected with pSiO_2_-COO-DEX had less vitritis and better vitreous clarity than either BSS intervened eyes (*p* = .0024, GEE) or free Dex intervened eyes (*p* = .0007, GEE). No neovascularization was noted on the medullary rays or elsewhere (Figure 3, pSiO_2_-COO-DEX 4wks–11wks) in this group. Along the disease course and at the last examination (week 11), the medullary ray elevation and distortion were severe in the BSS and free DEX treated eyes with a PVR grading of 4 or above. In contrast, the eyes treated with pSiO_2_-COO-DEX had only minimal surface wrinkling of the medullary ray. Overall, PVR severity was significantly less than either BSS intervened eyes (*p* < .0001, GEE) or free Dex intervened eyes (*p* = .0102, GEE). A retina/RPE atrophy area corresponding to the retinal bleb was noted (Figure 3, pSiO_2_-COO-DEX 11wks).

Due to the cloudy vitreous and medullary ray distortion, the eyes in the groups of free DEX and BSS became difficult for FA and OCT imaging. The OCT of the eyes injected with pSiO_2_-COO-DEX demonstrated early retinal elevation and later retinal atrophy at the retinal bleb area (Figure 4).

### Clinical and pathological difference between subretinal Matrigel injection at the medullary ray and subretinal VEGF/Matrigel injection

**Figure 6.**
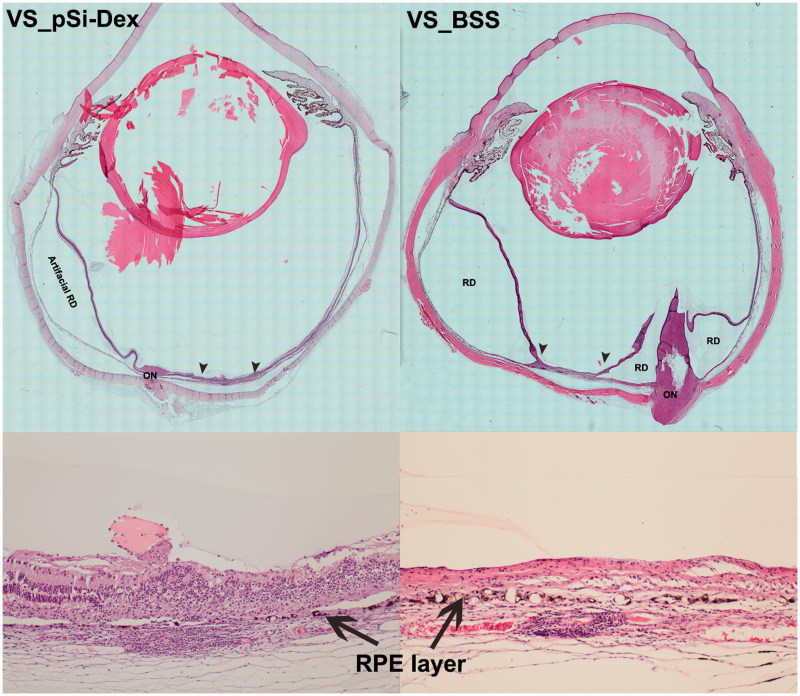
Histological analysis. The two images in the left column are from an eye treated with intravitreal pSiO_2_-COO-DEX (pSi-Dex) while the two images on the right column are from an eye treated by intravitreal BSS as control. In both eyes, the subretinal Matrigel + VEGF was injected in the visual streak (VS). The two images at the bottom are 10× light microscopy images corresponding to the area between the two arrowheads in the montages above. The montage VS_pSiO_2_-COO-DEX (VS_pSi-Dex) displays the injection site (between two arrowheads) next to the optic nerve (ON) and artificial retinal detachment (RD) from histological processing, evidenced by detachment between the RPE and choroid instead of neuroretinal detachment. In contrast, the montage VS_BSS demonstrates prominent proliferation of the optic nerve (ON) protruding into the vitreous, causing multiple pathological retinal detachments (RD). The RD inferior to the injection site is pathological RD with stiff retina and retinal gliosis. The magnified injection area shows RPE (arrows) damage and irregular proliferation with overlying retina atrophy and disorganization (Bottom images).

The aqueous flare score (0–4), (Jabs et al., 2005) aqueous cell score (0–4), (Jabs et al., 2005) and the vitreous clarity score (0–8) (Madow et al., 2011) from the clinical exams were summed to create an inflammatory score for each group. Since free Dex intervened eyes were similar to the BSS intervened eye as illustrated above (*p* = .59) for inflammatory reaction, those two groups were pooled to form the subretinal VEGF/Matrigel group for comparison with the group of subretinal Matrigel only injection at the medullary ray. GEE revealed that the addition of VEGF caused significantly more inflammation (*p* = .0044, GEE, Figure 5 left panel). PVR formation and scores were significantly more in the eyes injected with VEGF/Matrigel at the visual streak (*p* = .0015, GEE, Figure 5 right panel). However, no CNV was detected in any eye of the current study by either clinical exams or careful histological evaluation (Figure 6). Figure 6 confirmed PVR formation and efficacy of the pSiO_2_-COO-DEX delivery system.

## Discussion

Efforts to find treatments for eye diseases are no longer only focused on new drug development but also on various novel delivery systems or formulations of FDA approved drugs. For example, Retisert® (fluocinolone acetonide intravitreal implant), Ozurdex® (dexamethasone intravitreal implant), and Iluvien® (fluocinolone acetonide intravitreal implant) have been developed for ophthalmic use. A drug delivery system is often developed to target an array of diseases; however, a given drug is often approved by the FDA for a specific disease, then expanded to treat other diseases after a series of clinical trials (such as Ozurdex: https://www.drugs.com/history/ozurdex.html). This is because different diseases have different extents and severities of pathologies, such as inflammation or VEGF overexpression, which may need a specific window of drug concentration or potency to suppress. This is illustrated by a recent publication reporting that patients with more severe diabetic macular edema and poorer visual acuity responded better to the more potent aflibercept than to bevacizumab, while patients with better baseline visual acuity responded equally to both the drugs (Wells et al., 2015; Heier et al., 2016; Wells et al., 2016a,b). In addition, it is important to realize that higher concentrations will not always yield better pharmacological effects even when the concentration is within the safe range. A previous study demonstrated that 1 µg/mL or higher of dexamethasone promotes proliferation of retinal pigment epithelium *in vitro* (Pena et al., 1994). The dexamethasone level provided by the current pSi delivery system may be more suitable for PVR prevention than Ozurdex considering that the median time to develop PVR is about two months (Mietz & Heimann, 1995). Comparing this pSi-Dex delivery system with Ozurdex, one will note that Ozurdex provides retina dexamethasone level about 1110 ng/g within the period of two months (Chang-Lin et al., 2011) while this pSi-Dex system provide tapering drug concentration from 6 to 600 ng/mL within the same period (Hou et al., 2016). Dexamethasone has an EC50 of 3.4 ng/mL and an EC90 of 39 ng/mL (Jaffuel et al., 2001).

The current DEX-loaded porous silicon delivery system seems effective in suppressing PVR formation induced by subretinal injection of Matrigel and VEGF at the visual streak. This is very encouraging because the current study differs from the previous pretreatment design where the model disease was induced after medication application. A pretreatment design is to evaluate the therapeutic duration of the drug/delivery system (Hou et al., 2016). The current study was a real treatment study in which the disease model was induced prior to administration of the therapeutics. Such a design often needs higher drug concentrations or stronger potency to show pharmacological effect (El Bradey et al., 2004). This type of real treatment study is imperative for the evaluation of a drug delivery system in addition to the evaluation of its therapeutic duration. If a drug delivery system is only good for maintenance treatment, an induction treatment has to be provided (Teoh et al., 2012), which would impose more stress and economic burden on the patient

In the current study, subretinal injection of Matrigel at the medullary ray caused delayed vitritis, which started in the 2nd week and was active for about four weeks before regression. The inflammation originated around the injection site. There was hypofluorescence corresponding to the bleb in early phase FA, then hyperfluorescence in the late phase FA due to fluorescein pooling in the subretinal fluid. The OCT demonstrated subretinal fluid and a dome-shaped retinal detachment. However, no characteristic CNV leaking was observed in FAs and light microscopy did not identify any subretinal neovascularization. These observations were different from the previous report that used Matrigel at the same injection site (Qiu et al., 2006). The study by Qiu et al. observed similar CNV formation from both Matrigel and Matrigel plus VEGF injections, suggesting Matrigel, rather than the exogenous VEGF, attributed to the CNV formation. The dominant finding from the current study was the inflammation. There are several pathways involved in the development of PVR; however, ocular inflammation is the hallmark of PVR formation (Yoshida et al., 2017). Inflammation can cause the blood-retina barrier to breakdown. This breakdown allows cells, such as macrophages; growth factors, such as PDGF and TGF_β; or fibronectin to leak into the vitreous and cause cell migration and proliferation at the interface of the retina and vitreous (Bochaton-Piallat et al., 2000). Matrigel contains laminin, collagen IV, entactin, heparin sulfate proteoglycan, and growth factors. Therefore, it is logical that immunogenic inflammation was involved because of foreign proteins in the Matrigel. It is known that the rabbit is not an ideal CNV model by the standard laser breach of the Bruch’s membrane and accompanying inflammatory change (Ishibashi et al., 1987; Frank et al., 1989; Ishida et al., 1999; Campochiaro, 2000; Grossniklaus et al., 2010). The histology from the current study demonstrated proliferation and defects of the RPE cells. The RPE damage from the Matrigel-promoted inflammation was the reason for the late phase fluorescein pooling in the subretinal fluid shown in the current study. It is known that the chance of finding CNV in the subretinal fibrovascular tissue by histology is much higher than by angiographic identification (approximately 90% vs. 40% in primate laser-induced CNV model) (Grossniklaus et al., 2010). It is possible that in our study, we might miss the neovascularization between the abnormal RPE layer and the overlying retina tissue. However, CNV leakage during fluorescein angiography described by Qiu et al. was not present in the current study. Instead of a CNV model, subretinal injection of Matrigel at the medullary ray may serve as a model of inflammatory retina edema. The subretinally trapped Matrigel caused long-lasting inflammation. At eight weeks, inflammation in the vitreous around the bleb is clearly identifiable on OCT and subretinal fluid persists with hyperfluorescence in late phase FA (Figure 2). These features make this disease model promising for the screening of pharmacological therapeutics.

In addition to the subretinal injection of Matrigel at medullary ray, Matrigel plus VEGF was subretinally injected at the visual streak, the area equivalent to the human macula. The injection induced an inflammation (delayed vitritis) associated PVR model. In the second week after the subretinal injection, inflammation appeared in the anterior chamber and vitreous and lasted along with progressive proliferation and retinal neovascularization on the medullary ray, until the end of the study. This model is different from the published works by Kimura et al. or Ni et al. in which a recombinant basic fibroblast growth factor (FGF) was subretinally injected and CNV was induced in 83% lesions or 100% lesions by two weeks (Kimura et al., 1995; Ni et al., 2005). Both studies used FGF, instead of VEGF, along with a delivery system, either gelatin microspheres (Kimura et al., 1995) or heparin-sepharose beads. The difference between the current study and these two studies may come from sustained presence of the growth factor and chronic inflammation induced by the microspheres or beads themselves. Rabbit retina is very thin and fragile due to the lack of retinal vessels. The injected growth factor may slowly diffuse back through the retinotomy into the vitreous and cause inflammation associated PVR as seen in the current study. This interpretation is also supported by Ni et al. study that used a cocktail of FGF and lipopolysaccharide (LPS), but no obvious vitritis was reported. The inflammatory LPS might have bonded to the beads and remained subretinal.

The PVR model in this study was consistent and successful. The injection was performed under direct view of a surgical microscope and delivered by a Hamilton syringe loaded on a dispenser to ensure accuracy and repeatability. The retina and vitreous pathological evolution towards PVR can be monitored clearly using clinical instruments such as a slit-lamp, indirect ophthalmoscope, fluorescein angiography, and OCT to provide quantitative data for the evaluation of drug efficacy. This model also takes longer than the RPE cell injection model to reach its climax, which is advantageous for the screening of anti-proliferation and anti-angiogenesis compounds.

In summary, sustained delivery of dexamethasone from porous silicon particles was effective to inhibit PVR formation following a single intravitreal injection upon the presentation of inflammation and proliferation. Intravitreal free dexamethasone may have retarded PVR formation to some extent but not significantly, which highlights the importance of sustained drug presence at the disease site. This intervention was a true treatment study on a more realistic disease (PVR) model characterized by inflammation-driven proliferation and VEGF induced angiogenesis, resembling diabetic retinopathy and diabetic PVR. This single delivery strategy using the pSiO_2_-COO-DEX may be used as a perioperative adjunct pharmacological therapy for the prevention of PVR reoccurrence after vitreoretinal surgery. In addition, this PVR model from the subretinal injection of Matrigel and VEGF seems to be chronic and may be a better PVR model for the screening of therapeutics against proliferation.

## Supplementary Material

IDRD_Cheng_et_al_Supplemental_Content.zip

## References

[CIT0001] Agawa T, Usui Y, Wakabayashi Y, et al (2014). Profile of intraocular immune mediators in patients with age-related macular degeneration and the effect of intravitreal bevacizumab injection. Retina J Ret Vit Dis 34:1811–8. 10.1097/IAE.0000000000000157 24801651

[CIT0002] Agrawal RN, He S, Spee C, et al (2007). *In vivo* models of proliferative vitreoretinopathy. Nat Protoc 2:67–77. 17401340 10.1038/nprot.2007.4

[CIT0003] Arimura S, Takamura Y, Miyake S, et al (2016). The effect of triamcinolone acetonide or bevacizumab on the levels of proinflammatory cytokines after retinal laser photocoagulation in pigmented rabbits. Exp Eye Res 149:1–7. 27296072 10.1016/j.exer.2016.06.004

[CIT0004] Bakri SJ, Omar AF, Iezzi R, Kapoor KG. (2016). Evaluation of multiple dexamethasone intravitreal implants in patients with macular edema associated with retinal vein occlusion. Retina J Ret Vit Dis 36:552–7. 10.1097/IAE.0000000000000750 26418442

[CIT0005] Banerjee PJ, Quartilho A, Bunce C, et al (2017). Slow-release dexamethasone in proliferative vitreoretinopathy. Ophthalmology 124:757–67. 28237428 10.1016/j.ophtha.2017.01.021

[CIT0006] Bochaton-Piallat ML, Kapetanios AD, Donati G, et al (2000). TGF-beta 1, TGF-beta receptor II and ED-A fibronectin expression in myofibroblast of vitreoretinopathy. Invest Ophthalmol Vis Sci 41:2336–42. 10892881

[CIT0007] Browning AC, Chung AK, Ghanchi F, et al (2005). Verteporfin photodynamic therapy of choroidal neovascularization in angioid streaks: one-year results of a prospective case series. Ophthalmology 112:1227–31. 15921757 10.1016/j.ophtha.2005.02.011

[CIT0008] Campochiaro PA. (2000). Retinal and choroidal neovascularization. J Cell Physiol 184:301–10. 10911360 10.1002/1097-4652(200009)184:3<301::AID-JCP3>3.0.CO;2-H

[CIT0009] Campochiaro PA, Hafiz G, Mir TA, et al (2016). Pro-permeability factors in diabetic macular edema; the diabetic macular edema treated with ozurdex trial. Am J Ophthalmol 168:13–23. 27130369 10.1016/j.ajo.2016.04.017 PMC5482180

[CIT0010] Chang-Lin JE, Attar M, Acheampong AA, et al (2011). Pharmacokinetics and pharmacodynamics of a sustained-release dexamethasone intravitreal implant. Invest Ophthalmol Vis Sci 52:80–6. 20702826 10.1167/iovs.10-5285

[CIT0011] Cheng L, Anglin E, Cunin F, et al (2008). Intravitreal properties of porous silicon photonic crystals: a potential self-reporting intraocular drug-delivery vehicle. Br J Ophthalmol 92:705–11. 18441177 10.1136/bjo.2007.133587 PMC2666262

[CIT0012] Cheng LY, Hostetler K, Valiaeva N, et al (2010). Intravitreal crystalline drug delivery for intraocular proliferation diseases. Invest Ophthalmol Vis Sci 51:474–81. 19696179 10.1167/iovs.09-3672 PMC2869063

[CIT0013] El Bradey M, Cheng LY, Bartsch DU, et al (2004). Preventive versus treatment effect of Ag3340, a potent matrix metalloproteinase inhibitor in a rat model of choroidal neovascularization. J Ocul Pharmacol Ther 20:217–36. 15279727 10.1089/1080768041223657 PMC1360230

[CIT0014] El-Ghrably IA, Saad A, Dinah C. (2015). A novel technique for repositioning of a migrated ILUVIEN((R)) (Fluocinolone Acetonide) implant into the anterior chamber. Ophthalmol Ther 4:129–33. 26199036 10.1007/s40123-015-0035-1 PMC4675734

[CIT0015] Frank RN, Das A, Weber ML. (1989). A model of subretinal neovascularization in the pigmented rat. Curr Eye Res 8:239–47. 2468453 10.3109/02713688908997565

[CIT0016] Grossniklaus HE, Kang SJ, Berglin L. (2010). Animal models of choroidal and retinal neovascularization. Prog Retin Eye Res 29:500–19. 20488255 10.1016/j.preteyeres.2010.05.003 PMC2962694

[CIT0017] Heier JS, Bressler NM, Avery RL, et al (2016). Comparison of aflibercept, bevacizumab, and ranibizumab for treatment of diabetic macular edema extrapolation of data to clinical practice. Jama Ophthalmol 134:95–9. 26512939 10.1001/jamaophthalmol.2015.4110

[CIT0018] Hou H, Huffman K, Rios S, et al (2015). A novel approach of daunorubicin application on formation of proliferative retinopathy using a porous silicon controlled delivery system: pharmacodynamics. Invest Ophthalmol Vis Sci 56:2755–63. 25829415 10.1167/iovs.15-16526 PMC4416660

[CIT0019] Hou H, Nieto A, Ma F, et al (2014). Tunable sustained intravitreal drug delivery system for daunorubicin using oxidized porous silicon. J Control Release 178:46–54. 24424270 10.1016/j.jconrel.2014.01.003 PMC3951847

[CIT0020] Hou HY, Wang CY, Nan KH, et al (2016). Controlled release of dexamethasone from an intravitreal delivery system using porous silicon dioxide. Invest Ophthalmol Vis Sci 57:557–66. 26882530 10.1167/iovs.15-18559 PMC4758302

[CIT0021] Ishibashi T, Miller H, Orr G, et al (1987). Morphological observations on experimental subretinal neovascularization in the monkey. Invest Ophthalmol Vis Sci 28:1116–30. 2439474

[CIT0022] Ishida K, Yoshimura N, Mandai M, Honda Y. (1999). Inhibitory effect of TNP-470 on experimental choroidal neovascularization in a rat model. Invest Ophthalmol Vis Sci 40:1512–9. 10359334

[CIT0023] Jabs DA, Nussenblatt RB, Rosenbaum JT, Nomenclatu SU. (2005). Standardization of uveitis nomenclature for reporting clinical data. Results of the first international workshop. Am J Ophthalmol 140:509–16. 16196117 10.1016/j.ajo.2005.03.057 PMC8935739

[CIT0024] Jaffuel D, Roumestan C, Balaguer P, et al (2001). Correlation between different gene expression assays designed to measure trans-activation potencies of systemic glucocorticoids. Steroids 66:597–604. 11322967 10.1016/s0039-128x(00)00235-x

[CIT0025] Kimura H, Sakamoto T, Hinton DR, et al (1995). A new model of subretinal neovascularization in the rabbit. Invest Ophthalmol Vis Sci 36:2110–9. 7657549

[CIT0026] Liu F, Ding XY, Yang Y, et al (2016). Aqueous humor cytokine profiling in patients with wet AMD. Mol Vis 22:352–61. 27122966 PMC4842003

[CIT0027] Madow B, Galor A, Feuer WJ, et al (2011). Validation of a photographic vitreous haze grading technique for clinical trials in uveitis. Am J Ophthalmol 152:170–6. 21652026 10.1016/j.ajo.2011.01.058 PMC4556733

[CIT0028] Meyer LM, Schonfeld CL. (2013). Secondary glaucoma after intravitreal dexamethasone 0.7 mg implant in patients with retinal vein occlusion: a one-year follow-up. J Ocul Pharmacol Ther 29:560–5. 23480270 10.1089/jop.2012.0253

[CIT0029] Mietz H, Heimann K. (1995). Onset and recurrence of proliferative vitreoretinopathy in various vitreoretinal diseases. Br J Ophthalmol 79:874–7. 7488572 10.1136/bjo.79.10.874 PMC505285

[CIT0030] Ni M, Holland M, Jarstadmarken H, De Vries G. (2005). Time-course of experimental choroidal neovascularization in Dutch-Belted rabbit: clinical and histological evaluation. Exp Eye Res 81:286–97. 16129096 10.1016/j.exer.2005.01.027

[CIT0031] Nieto A, Hou H, Sailor MJ, et al (2013). Ocular silicon distribution and clearance following intravitreal injection of porous silicon microparticles. Exp Eye Res 116:161–8. 24036388 10.1016/j.exer.2013.09.001 PMC3873878

[CIT0032] Pardo-Lopez D, Frances-Munoz E, Gallego-Pinazo R, Diaz-Llopis M. (2012). Anterior chamber migration of dexametasone intravitreal implant (Ozurdex(R)). Graefes Arch Clin Exp Ophthalmol 250:1703–4. 21861084 10.1007/s00417-011-1802-x

[CIT0033] Pena RA, Jerdan JA, Glaser BM. (1994). Effects of TGF-beta and TGF-beta neutralizing antibodies on fibroblast-induced collagen gel contraction: implications for proliferative vitreoretinopathy. Invest Ophthalmol Vis Sci 35:2804–8. 8188474

[CIT0034] Qiu G, Stewart JM, Sadda S, et al (2006). A new model of experimental subretinal neovascularization in the rabbit. Exp Eye Res 83:141–52. 16579984 10.1016/j.exer.2005.11.014

[CIT0035] Ramu J, Yang Y, Menon G, et al (2015). A randomized clinical trial comparing fixed vs. pro-re-nata dosing of Ozurdex in refractory diabetic macular oedema (OZDRY study). Eye 29:1603–12. 26493038 10.1038/eye.2015.214 PMC5129797

[CIT0036] Rezar-Dreindl S, Sacu S, Eibenberger K, et al (2016). The intraocular cytokine profile and therapeutic response in persistent neovascular age-related macular degeneration. Invest Ophthalmol Vis Sci 57:4144–50. 27537264 10.1167/iovs.16-19772

[CIT0037] Sha O, Kwong WH. (2006–07). Postnatal developmental changes of vitreous and lens volumes in sprague–dawley rats. Neuroembryol Aging 4:183–8.

[CIT0038] Teoh SC, Ou XL, Lim TH. (2012). Intravitreal ganciclovir maintenance injection for cytomegalovirus retinitis: efficacy of a low-volume, intermediate-dose regimen. Ophthalmology 119:588–95. 22137552 10.1016/j.ophtha.2011.09.004

[CIT0039] Wang C, Hou H, Nan K, et al (2014a). Intravitreal controlled release of dexamethasone from engineered microparticles of porous silicon dioxide. Exp Eye Res 129:74–82. 25446320 10.1016/j.exer.2014.11.002 PMC4259850

[CIT0040] Wang CY, Hou HY, Nan KH, et al (2014b). Intravitreal controlled release of dexamethasone from engineered microparticles of porous silicon dioxide. Exp Eye Res 129:74–82. 25446320 10.1016/j.exer.2014.11.002 PMC4259850

[CIT0041] Wang JK, Huang TL, Su PY, Chang PY. (2015). An updated review of long-term outcomes from randomized controlled trials in approved pharmaceuticals for diabetic macular edema. Eye Sci 30:176–83. 27215008

[CIT0042] Wells JA, Glassman AR, Ayala AR, et al (2015). Aflibercept, bevacizumab, or ranibizumab for diabetic macular edema. N Engl J Med 372:1193–203. 25692915 10.1056/NEJMoa1414264 PMC4422053

[CIT0043] Wells JA, Glassman AR, Ayala AR, et al (2016a). Aflibercept, bevacizumab, or ranibizumab for diabetic macular edema two-year results from a comparative effectiveness randomized clinical trial. Ophthalmology 123:1351–9. 26935357 10.1016/j.ophtha.2016.02.022 PMC4877252

[CIT0044] Wells JA, Glassman AR, Jampol LM. (2016b). Association of Baseline Visual Acuity and Retinal Thickness With 1-Year Efficacy of Aflibercept, Bevacizumab, and Ranibizumab for Diabetic Macular Edema (vol 134, pg 127, 2016). JAMA Ophthalmol 134:469. 10.1001/jamaophthalmol.2015.4599 PMC5567793 26605836

[CIT0045] Xu Y, Tan CS. (2017). Safety and complications of intravitreal injections performed in an Asian population in Singapore. Int Ophthalmol 37:325–332. 27236451 10.1007/s10792-016-0241-4

[CIT0046] Yoshida S, Kobayashi Y, Nakao S, et al (2017). Differential association of elevated inflammatory cytokines with postoperative fibrous proliferation and neovascularization after unsuccessful vitrectomy in eyes with proliferative diabetic retinopathy. Opth 11:1697–1705. 10.2147/OPTH.S141821 PMC5614779 29033535

