# Supplementary figures and images for: New model of proliferative vitreoretinopathy in rabbit for drug delivery and pharmacodynamic studies
Source: Drug Deliv. 2018 Feb 20;25(1):600–10. doi: 10.1080/10717544.2018.1440664 (PMC6058613; doi:10.1080/10717544.2018.1440664)

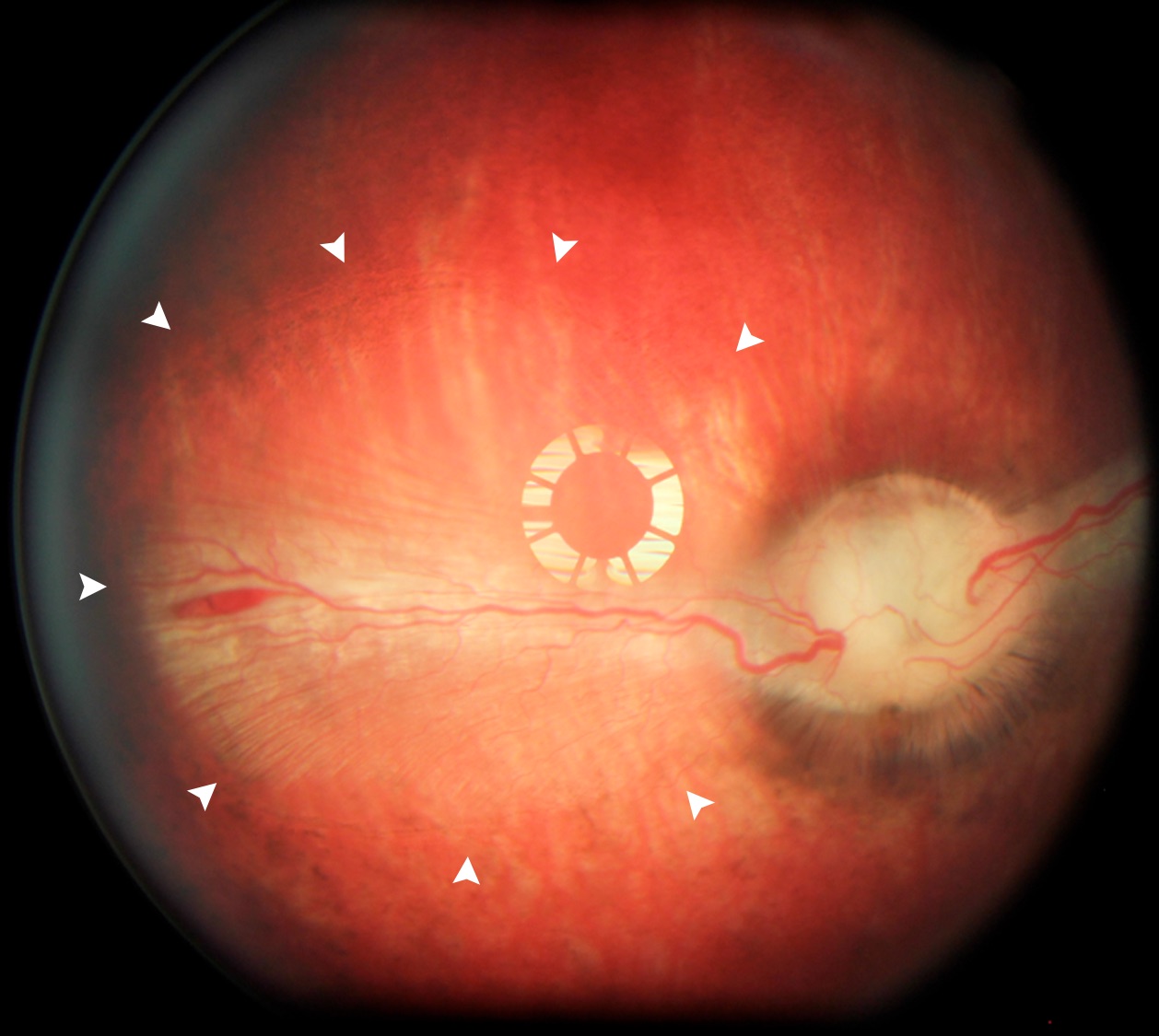

Supplement: IDRD_Cheng_et_al_Supplemental_Content.zip [file IDRD_A_1440664_SM4866.zip › Supplemental Figure 1.jpg]

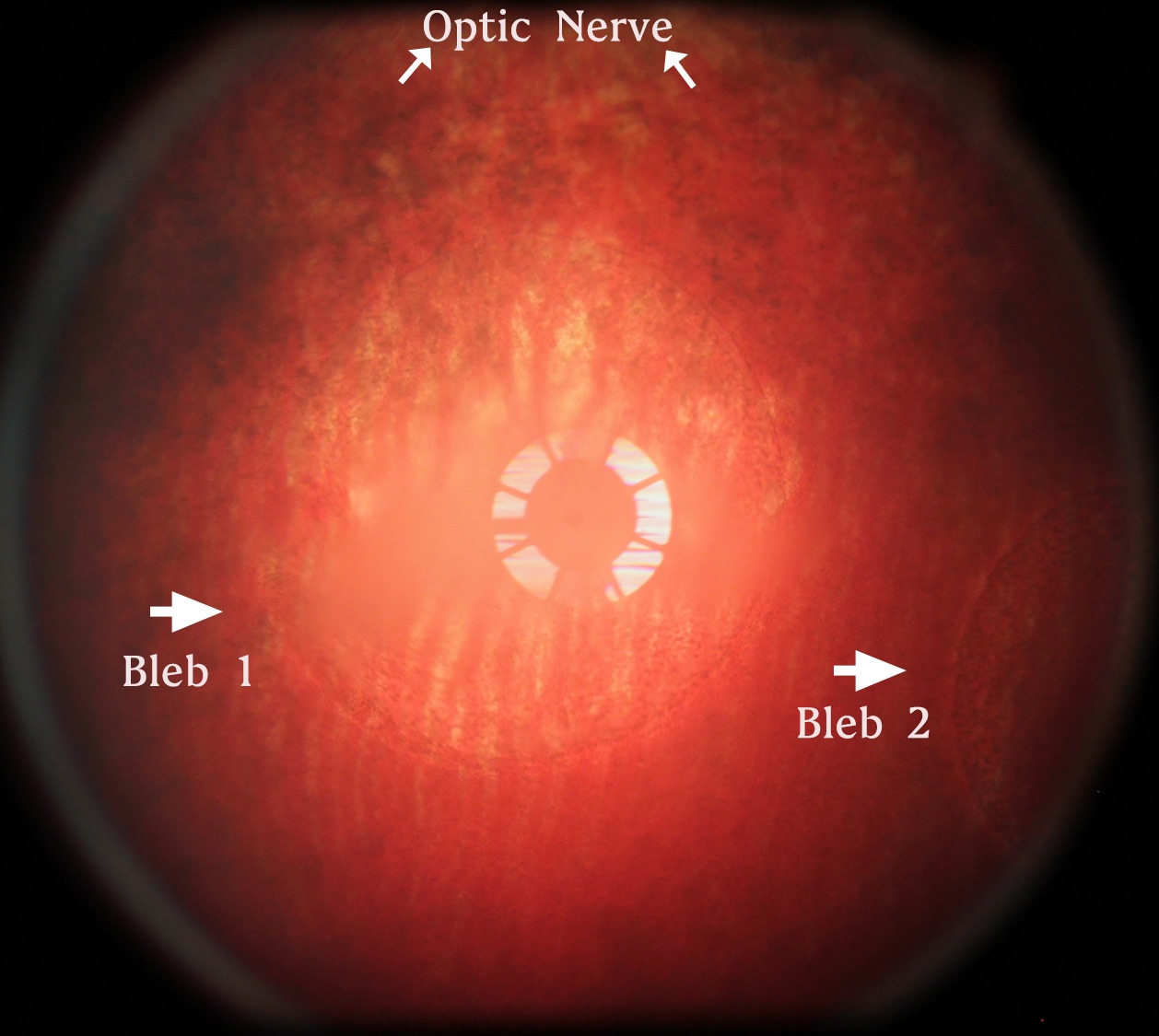

Supplement: IDRD_Cheng_et_al_Supplemental_Content.zip [file IDRD_A_1440664_SM4866.zip › Supplemental Figure 2.jpg]

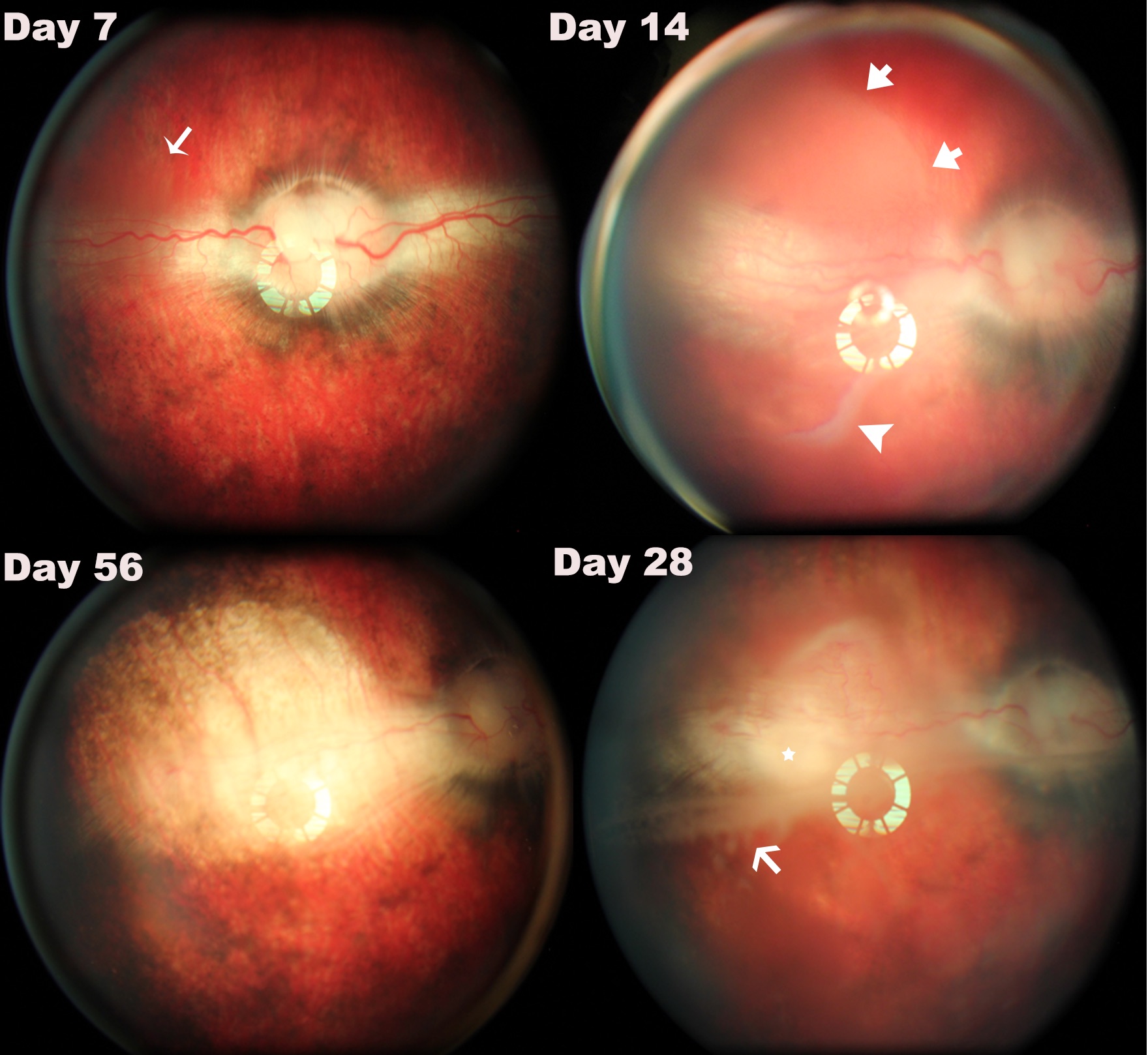

Supplement: IDRD_Cheng_et_al_Supplemental_Content.zip [file IDRD_A_1440664_SM4866.zip › Supplemental Figure 3.jpg]

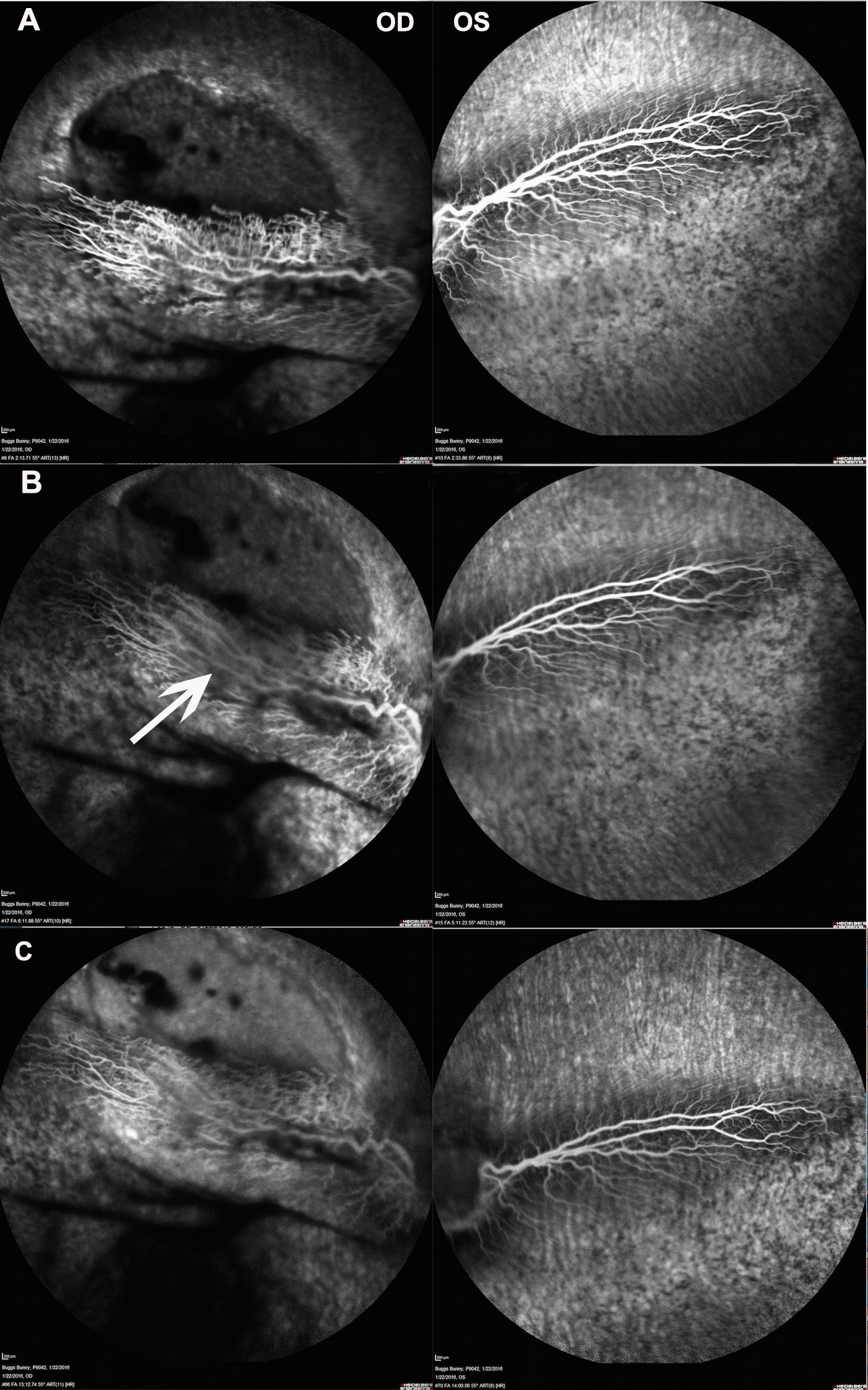

Supplement: IDRD_Cheng_et_al_Supplemental_Content.zip [file IDRD_A_1440664_SM4866.zip › Supplemental Figure 4.jpg]

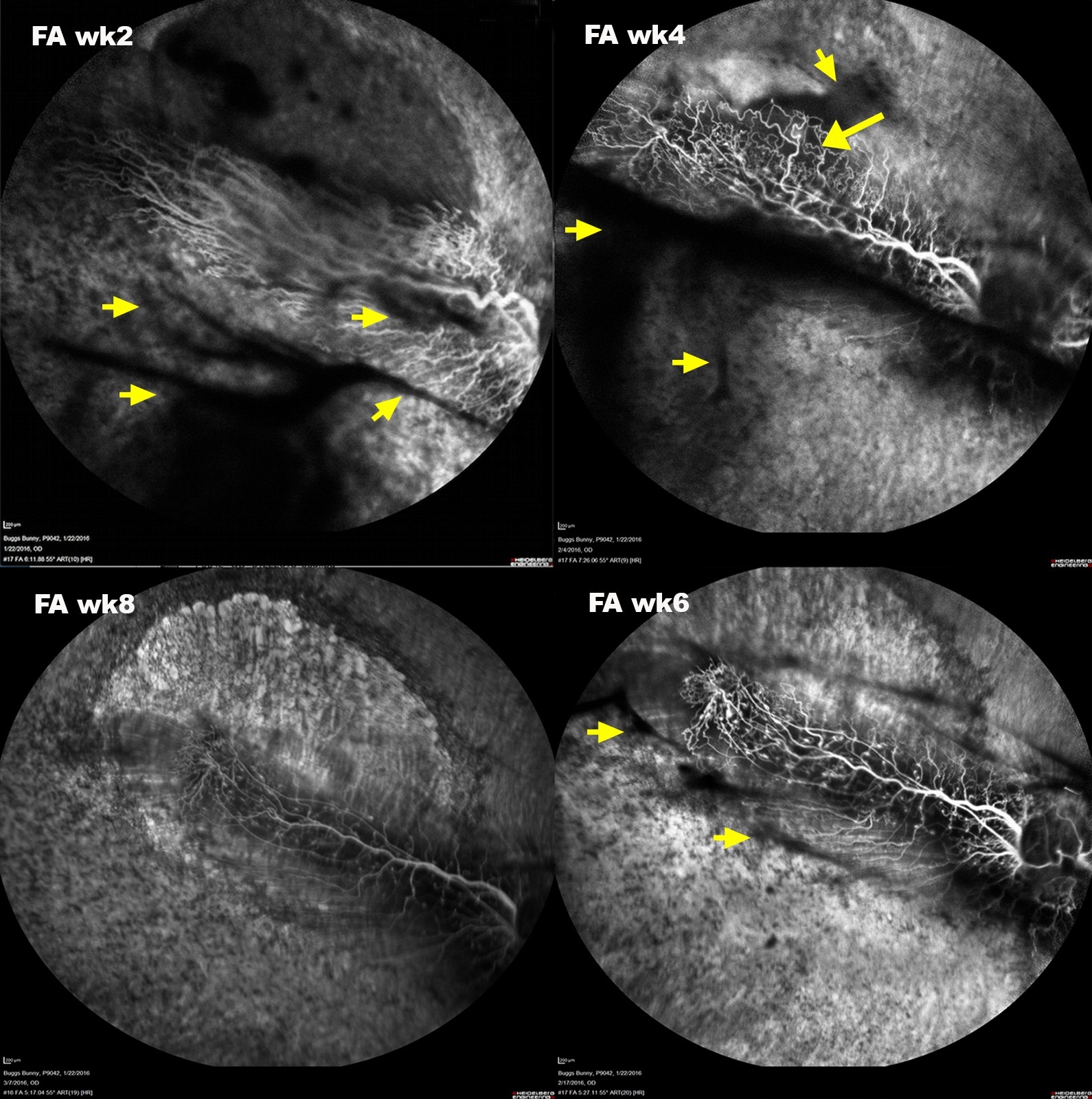

Supplement: IDRD_Cheng_et_al_Supplemental_Content.zip [file IDRD_A_1440664_SM4866.zip › Supplemental Figure 5.jpg]
